# Evolution of Elevated-Temperature Strength and Creep Resistance during Multi-Step Heat Treatments in Al-Mn-Mg Alloy

**DOI:** 10.3390/ma11071158

**Published:** 2018-07-07

**Authors:** G. S. Wang, K. Liu, S. L. Wang

**Affiliations:** 1 Key Laboratory of Electromagnetic Processing of Materials, Ministry of Education, Northeastern University, Shenyang 110819, China; wanggs@epm.neu.edu.cn (G.S.W.), 1770372@stu.neu.edu.cn (S.L.W.); 2 School of Materials Science and Engineering, Northeastern University, Shenyang 110819, China; 3 Department of Applied Science, University of Quebec at Chicoutimi, Saguenay, QC G7H 2B1, Canada

**Keywords:** Al-Mn-Mg alloy, dispersoids, heat treatment, elevated temperature, strength, creep resistance

## Abstract

The present work has systematically investigated the evolution of dispersoids and elevated-temperature properties including strength and creep resistance during various multi-step heat treatments in Al-Mn-Mg 3004 alloys. Results show that only the α-Al(MnFe)Si dispersoid is observed in the studied temperature range (up to 625 °C), and that it coarsens with increasing temperature to 500 °C, but dissolves at 625 °C. The evolution of elevated-temperature strength and creep resistance is greatly related to the temperature of each step during the multi-step heat treatments. Generally, lower temperature at the first-step heat treatment leads to higher properties, while the properties decrease with increasing temperature of last-step heat treatment. Suitable models have been introduced to explain the evolution of strength and the creep threshold stress at elevated-temperatures during the various heat treatments.

## 1. Introduction

Nowadays, the applications of dispersoid-strengthening aluminum alloys are increasing in the automotive and aerospace industries due to the formation of dispersoids that remain thermal-stable at elevated temperatures (250–350 °C) [1,2]. It is reported that coherent or semi coherent Al_3_M dispersoids can precipitate and remain thermal-stable up to 250–350 °C through the addition of transition and rare earth elements, such as by the individual or combined addition of Sc, Zr, and/or Er [3,4,5,6]. However, the high cost of these elements limits their wide application. 

In the present decade, dispersoids have also been reported to precipitate in Al-Mn 3xxx alloy during proper heat treatment; the precipitation behavior of dipsersoids has been fully investigated. It is reported that the yield strength of 3003 at room temperature (RT) after 375 °C/24 h has been improved to 80 MPa, compared with 52 MPa after 600 °C/24 h, due to the precipitation of dispersoids [7]. Recently, Liu et al., has systematically studied the evolution of dispersoids at relatively lower heat treatment temperatures (300–425 °C), and their influence on elevated-temperature properties in Al-Mn-Mg 3004 alloys [6,8,9,10,11,12]. It was found that high volume fraction and well-distributed α-Al(MnFe)Si dispersoids are observed after treatment at 375 °C for 48 h, leading to remarkable improvements in YS and creep resistance at elevated temperatures. On the other hand, it is widely reported the multi-heat treatments have a significant influence on the evolution of intermetallics and properties, as well as on recrystallization behavior, especially in Al-Mg alloys [13,14,15,16,17]. However, the heat treatment described in the above literature is limited to one-step at relatively low temperatures; limited literature has been published on the evolution of dispersoids and elevated-temperature properties during heat treatments at high temperature, as well as multi-step heat treatments in 3xxx alloys. 

On the other hand, creep resistance is considered to be one of the most significant parameters in alloy application at elevated temperatures [18,19,20]. Generally, the dislocation creep occurs in aluminum alloys at 250–350 °C, and the stress exponent and creep threshold stress have been well developed to explain the controlling mechanism during creep tests on aluminum alloys, especially in 1xxx alloys [21,22,23,24]. However, most of the literature focuses on nano-scale precipitates or particles, and the influence of bigger dispersoids (˃50 nm in diameter) on creep resistance has been less dealt with. Little work has been performed on the creep resistance in Al-Mn 3xxx alloys, and the evolution of creep threshold stress during the heat treatment is not fully studied.

Therefore, in order to fully investigate the evolution of dispersoids during the various heat treatments, and their influence on elevated-temperature strength and creep behavior, wide-temperature-range and multi-step heat treatments were applied to an Al-Mn-Mg 3004 alloy in the present work and the strength and creep behavior at 300 °C were measured and characterized. Meanwhile, suitable models are established to evaluate the relationship between dispersoids and elevated-temperature properties. 

## 2. Experimental

### 2.1. Alloy Preparation

AA3004 alloy was prepared with commercially pure Al (99.7%) and pure Mg (99.9%), Al-25%Fe, Al-50%Si and Al-25%Mn master alloys. The chemical composition of the AA3004 alloy is Al-1.2%Mn-1.1%Mg-0.6%Fe-0.25%Si, analyzed using an optical emission spectrometer (OES).

In each test, approximately 3 kg of material was prepared in a clay-graphite crucible using an electric resistance furnace. The temperature of the melt was maintained at ~750 °C for 30 min. The melt was degassed for 15 min and then poured into a permanent mold preheated at 250 °C. The dimensions of cast ingots were 30 mm × 40 mm × 80 mm.

### 2.2. Heat Treatment

According to our previous study [9], the onset precipitation temperature of dispersoids is around 340 °C, and the best conditions can be obtained at 375 °C/48 h in the range of 300–425 °C. Therefore, another two, higher temperatures (500 °C and 625 °C) were selected in the present work, and the condition of 375 °C/48 h was considered to be the base heat treatment in multi-step heat treatments. The heat treatments were performed in a programmable electric furnace with a temperature controller (±2 °C) and circulating air. The heating rate was set as 5 °C/min. After each step, the ingots were directly quenched into water at RT. More detail of heat treatments is shown in Table 1. 

### 2.3. Evolution of Alloy Properties

The precipitation behavior of dispersoids during precipitation treatment and thermal holding were evaluated by Electrical Conductivity (EC) and Yield strength (YS). EC was measured using a Sigmascope SMP10 electrical conductivity unit at RT, and the average value of 5 measurements was recorded for each sample. Mechanical properties (YS) were determined from compression tests performed on a Gleeble 3800 machine. Cylindrical specimens with a length/diameter ratio of 1.5 (i.e., 15 mm in length and 10 mm in diameter) were machined and tested at both RT and at an elevated temperature (300 °C) following the ASTM E9-89a standard. The total deformation of the specimens was set to 0.2, and the strain rate was fixed at 10^−3^ s^−1^. For the compression test at 300 °C, the specimen was heated to 300 °C with a heating rate of 2 °C/s, and held for 3 min to stabilize. An average value of YS was obtained from 3 tests.

In addition, creep tests were performed at 300 °C, and specimens were the same size as the Gleeble samples. Creep resistance was tested at a constant load of 38 MPa, while another two loads (28 and 32 MPa) were applied for the creep threshold stress and stress exponents. For each condition, 3 tests were performed to confirm the reliability of the results.

### 2.4. Microstructure Observation

The microstructural features, including the intermetallics, dispersoids, and grain structures for different conditions, were observed by optical and electron microscopes. To reveal the dispersoids clearly, the polished samples were etched in 0.5% HF for 30 s. A scanning electron microscope (SEM, JSM-6480LV) equipped with an energy dispersive X-ray spectrometer (EDS) and electron backscatter diffraction (EBSD) was used to examine the intermetallics and grain structure of the alloy under different conditions. A transmission electron microscope (TEM, JEM-2100) operated at 200 kV was used to observe the distribution of the dispersoids. The thickness of the TEM sample was measured with electron energy loss spectroscopy (EELS). The size and number density of dispersoids were measured using Clemex PE 4.0 image analysis software with the TEM images. In this study, the volume fraction of the particle free zone (PFZ) was converted from the area fraction of the PFZ measured by image analysis from optical images according to the Delesse’s principle [25,26], while the volume fraction of dispersoids was calculated according to the model introduced in the literature [27] and shown in Equation (1):

(1) $$V_{V}{=A}_{A}\frac{\bar{\mathrm{KD}}}{\bar{\mathrm{KD}}+t}(1-A_{PFZ})$$

where $\bar{D}$ is the average equivalent diameter of dispersoids; t is the TEM foil thickness; A_A_ is the area percentage of dispersoids from TEM observation; *A_PFZ_* is the area percentage of the particle free zone (PFZ) from OM measurements; and $\bar{K}$ is the average shape factor of dispersoids [27]. 

## 3. Results and Discussion

### 3.1. Precipitation of Dispersoids during the Heat Treatment

**Figure 1:**
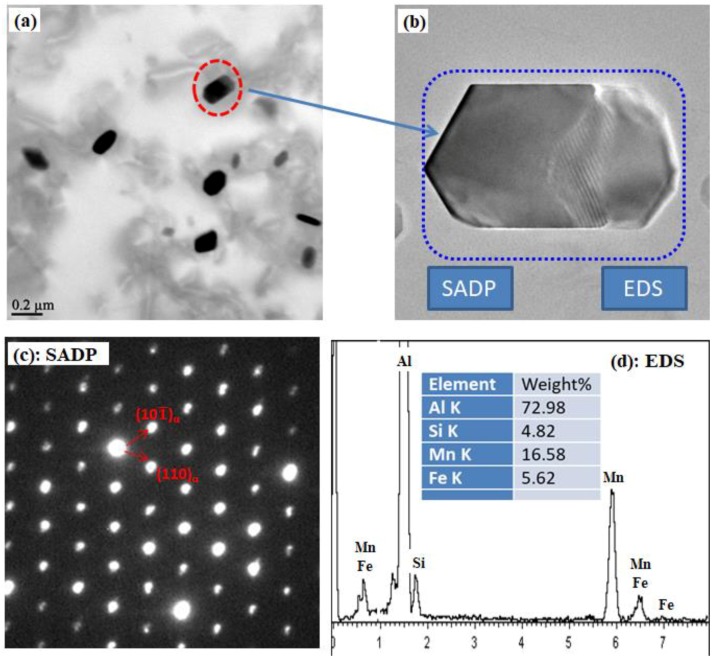
Dispersoids after A6 and its selected area diffraction pattern (SADP), as well as the EDS results: (**a**) Distribution of dispersoids (**b**) Dispersoid particle; (**c**) SADP of dispersoid in (b); (**d**) EDS result of dispersoid in (b).

Although the formation of Al_6_(MnFe) dispersoids has been reported when treated at 600 °C in 3103 alloy [28] and at 555 °C in 5083 [15], dispersoids after all the heat treatments performed in the present work are identified as α-Al(MnFe)Si, even at 600 °C. As an example, Figure 1 shows the dispersoids precipitated after A6 with the highest “H” temperature (625 °C) and the TEM-EDS result. As shown in Figure 1a, the dispersoids can be present with different morphologies, such as cubic and platelet-like. Figure 1b is the enlarged image of one particle in Figure 1a, while Figure 1c shows its selected area diffraction pattern (SADP) and TEM-EDS results in Figure 1d. As shown in Figure 1c, the dispersoids precipitated after A6 are determined as cubic α-Al(MnFe)Si rather than orthorhombic Al_6_(MnFe) dispersoids from the SADP of dispersoids in the [$\bar{1}$1$\bar{1}$] direction [9,29,30]. Meanwhile, the TEM-EDS result (Figure 1d) shows that all the dispersoids are composed of Mn, Fe, and Si, identifying the dispersoids as α-Al(MnFe)Si. One of the likely reasons for free of Al_6_(MnFe) dispersoids in the present work is the higher Si content in experimental 3004 alloy (0.25%) than the 3103 alloy in the literature (0.05%), which promotes the precipitation of α-Al(MnFe)Si dispersoids. Therefore, all the dispersoids talked about in the following part are referred to as α-Al(MnFe)Si dispersoids.

**Figure 2:**
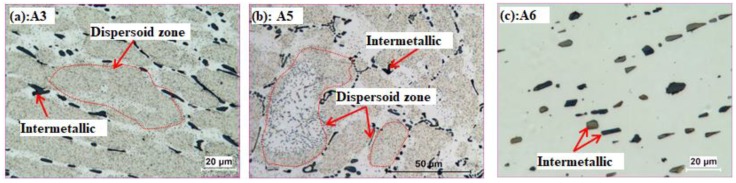
Distribution of dispersoids after single-step heat treatment: (**a**) A3, (**b**) A5 and (**c**) A6.

Figure 2 shows the distribution of dispersoids under single heat treatments (A3, A5 and A6). It shows that the finer α-Al(MnFe)Si dispersoids are uniformly precipitated after A3 (375 °C/48 h). Only a low volume fraction of PFZ can be observed surrounding the intermetallic particles (interdentritic zone) after A3, which can be attributed to the depletion of Mn near the intermetallics due to the formation of primary Al_6_(MnFe) and α-Al(MnFe)Si intermetallics [31]. By increasing the temperature to 500 °C (Figure 2b), the size of the dispersoids increases. Meanwhile, PFZ begins to form in the center of dendrite cells, where some much bigger dispersoids present, indicating that a coarsening of dispersoids happens at 500 °C compared with 375 °C. However, few dispersoids can be observed with by increasing temperature to 625 °C. As shown in Figure 2c, only fragmented intermetallics are present in the Al matrix after A6 (625 °C/4 h). In the literature, it is reported that the α-Al(MnFe)Si dispersoids begin to dissolve at 600 °C [27,28,32]; hence, it is reasonable for the dispersoids to dissolve into the matrix at 625 °C.

**Figure 3:**
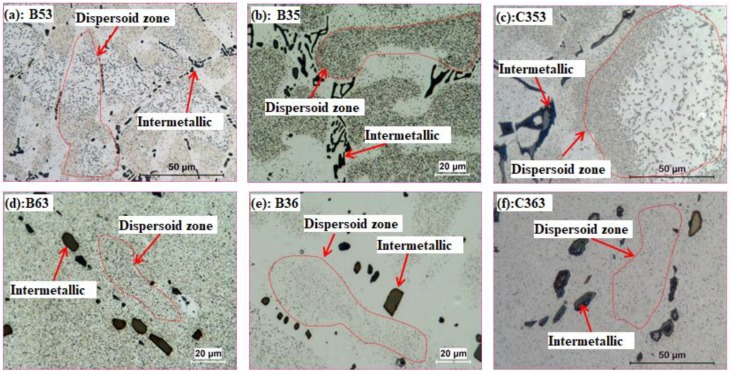
Distribution of dispersoids after two-step and three-step heat treatment: **(a**) B53, (**b**) B35, (**c**) C353, (**d**) B63, (**e**) B36 and (**f**) C363.

The distribution of dispersoids after two- and three-step heat treatments is shown in Figure 3 Generally, coarsening of dispersoids happens with treatment at 500 °C, either under two- or three-step heat treatment compared with A3. The volume fraction of PFZ increases after two- or three-step heat treatment at 500 °C. As shown in Table 2, the volume fraction of PFZ slightly increases from 32 vol. % in A5 to 37 vol. % in B35, but rapidly to 45 vol. % in C353, and further to 55 vol. % in B53. The slight increase in PFZ from A5 to B35 is mainly due to the dispersoids coarsening, and more PFZ is shown in the interdendrite zone (Figure 3b). However, it was noted that the volume fraction of bigger dispersoids in the center of dendrite cells increases after B53 (Figure 3a) and C353 (Figure 3c) due to the inverse segregation of Mn and Fe [28], leading to the higher volume fraction of PFZ. 

However, the distribution of dispersoids after treatment at 625 °C is more complicated. As shown in Figure 3d, the dispersoids are observed to distribute everywhere, and PFZ disappeared after B63, which is probably attributed to uniform solute Mn atoms in the matrix after 625 °C for 4 h (homogenization). A similar phenomenon takes place after C363 (Figure 3f), in which the dispersoids first precipitate in the first step at lower temperature (375 °C), and then dissolve into the matrix in the second step at higher temperature (625 °C), followed by the re-precipitate of uniformly-distributed dispersoids in the third step at lower temperature (375 °C). However, it seems that the volume fraction of dispersoids after C363 is a little lower than B63, which is probably caused by decreasing solute Mn concentration from incompletely dissolved dispersoids in the second step, which is also shown in Figure 3e. It is observed that a low volume fraction of dispersoids still remains after B36 (Figure 3e) due to the incompletely dissolved dispersoids.

**Figure 4:**
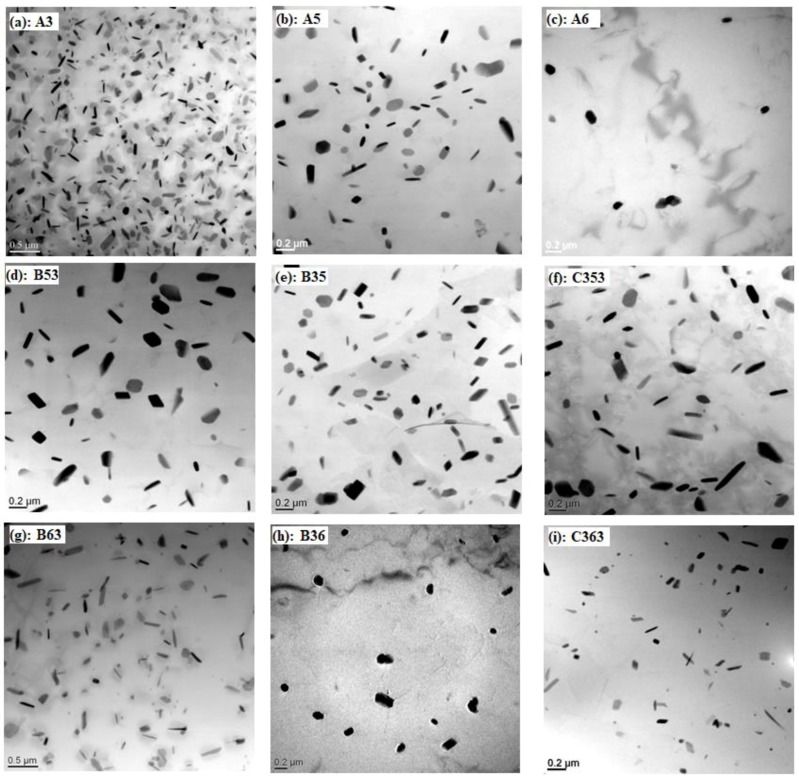
Distribution of dispersoids during heat treatment under TEM observation: (**a**) A3**,** (**b**) A5, (**c**) A6, (**d**) B53, (**e**) B35, (**f**) C353, (**g**) B63, (**h**) B36 and (**i**) C363.

In order to show more detail about the dispersoids, TEM was performed; the distribution of dispersoids after various heat treatment is shown in Figure 4. Similar to the distribution of dispersoids shown in Figure 2 and Figure 3, the highest volume fraction of dispersoid precipitates in a single treatment at lower temperature (A3 in Figure 4a) and the coarsened dispersoids precipitate after heat treatment at 500 °C (A5 in Figure 4b, B53 in Figure 4d, and C353 in Figure 4f). As shown in Table 2, the equivalent diameter of dispersoids after heat treatment at 500 °C is in the range of 107–115 nm, which is much higher than in A3 (67 nm). The volume fraction of dispersoids varies with heat treatment, especially during multi-step heat treatments at high temperature (600 °C). The highest was measured in B35, which is 1.67 vol. %, which can be attributed to the first step heat treatment at lower temperature, in which the dispersoids have been fully precipitated with a relatively low coarsening rate of dispersoids at high temperature. However, some dispersoids are still observed after treatment at 625 °C for 4 h (A6 in Figure 4c), but the volume fraction is very low, just 0.27 vol. % (Table 2), which is different with the optical observation in Figure 2c. Dipsersoids are also observed after B36 with a little higher volume fraction (0.51 vol. %) than A6, which agrees with the results obtained from Figure 2 and Figure 3. Furthermore, an interesting thing happens in condition B63 and C363, namely, a high volume fraction of dispersoids presents after etching in optical microscope observation (Figure 3d,f) but a much lower volume fraction of dispersoids was in TEM observation (Figure 4g,i). The lower volume fraction after B63 and C363 under TEM is probably related to the phase transformation of intermetallics at high temperature. It was reported that Al_6_(MnFe) would transform to α-Al(MnFe)Si intermetallics at 600 °C, and the Mn/Fe ratio would increase with holding time [33]. Therefore, partial Mn atoms diffuse to α-Al(MnFe)Si intermetallics during the phase transformation at high temperature, leading to a decreased solute Mn concentration in matrix. Therefore, the drive force for the precipitation of dispersoids decreases with the decreasing supersaturation of Mn in matrix, but Mn is uniformly distributed. Therefore, dispersoids precipitate uniformly due to the homogenized Mn atoms in the matrix, but with a lower volume fraction from lower Mn concentration in Al matrix in the last step at lower temperature (375 °C); this presents as uniform distributed dispersoids in optical microscope without PFZ.

### 3.2. Compression Yield Strength 

**Figure 5:**
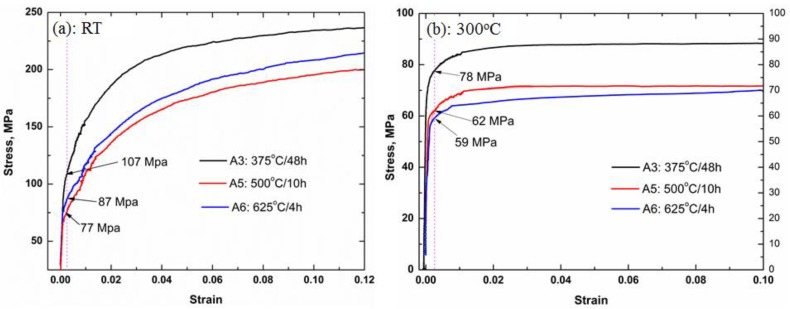
True strain-stress curves of alloy after A3, A5, and A6 at both RT (**a**) and 300 °C (**b**).

As shown in Figure 2, Figure 3 and Figure 4, great changes occur in the characteristics of dispersoids during the heat treatment, which will definitely affect the mechanical properties of alloys. Figure 5 shows the typical true strain-stress curves of the alloy after single step heat treatment (A3, A5 and A6) at both RT and 300 °C. 

As shown in Figure 5a, the compression YS at RT varies with A3, A5 and A6. The highest YS (108 MPa) is obtained in A3 due to the dispersoid strengthening from the highest volume fraction of dispersoids, and YS decreases with the coarsening and decreasing volume fraction in A5 (77 MPa), while the YS between A5 and C353 is similar. However, the YS of A6 is between A3 and A5 though with lowest volume fraction of dispersoids. This can be attributed to the strong solid-solution strengthening from Mn in Al matrix in A6 condition. On the other hand, due to the weakened solution-hardening effect at 300 °C (Figure 5b), YS of A6 is lower than A5, and further lower than A3, which is 59 MPa in A6 to 62 MPa in A5, and 78 MPa in A3, as shown in Figure 3b. The YS and EC of all heat treatments are listed in Table 3.

As shown in Table 3, EC is mainly controlled by the last step, which is similar to the literature [32]. For instance, EC of A3, B53, B63, C353, and C363 is similar, which is around 38%IACS. For, it can be observed that the evolution of YS decreases with increasing temperature, i.e. by the descending YS in A3, A5, and A6. Besides, the first-step temperature plays a significant role in YS in multi-steps heat treatment, in which that YS is higher with lower first-step temperature than with higher first-step temperature. This can be proven from the YS between B35 and B53, B36 and B63 in Table 3. 

In order to explain the evolution of YS at both RT and 300 °C, two principal processes involved during the heat treatment were considered in the present work: precipitation of dispersoids at lower temperature (375 °C and 500 °C) for dispersoid strengthening, and dissolution of dispersoids at high temperature (625 °C) for solid-solution strengthening. Therefore, the evolution of YS can be estimated with the synergetic effect of these two factors. 

According to the literature, the contribution of dispersoids to the YS of alloys (*σ*_D_) can be estimated with the Ashby-Orowan equation [7,34]:

(2) $$\sigma_{D}=\frac{0.84\mathrm{MGb}}{2\pi {\left(1-v\right)}^{1/2}r}\ln(\frac{\gamma }{b}){\left(\frac{2\pi }{3f}\right)}^{-1/2}$$

where *M* is the Taylor factor (2), *G* is the temperature-dependent matrix shear modulus (given as *G* = 25.4[1−0.5(*T*−300)/933] [35]), and *b*, *ν* *γ* and *f* are the burgers vector of dislocation (0.286 nm), the poison ratio (0.34), the radius, and the volume fraction of dispersoids in Table 2, respectively.

On the other hand, the solid-solution strengthening from Mn atoms is reported to have a linear relationship with solute Mn concentration, as [36]:

(3) $$\sigma_{\mathrm{Mn}}{=\mathrm{HC}}^{\alpha }$$

where H and α are constant, i.e., 18.35 and 0.9 for solute Mn atom under at *RT*, or 10 and 0.8 at 300 °C under the conditions applied in the present work (strain rate at 10^−3^ s^−1^ and total strain at 0.2%) [1,36,37,38]. *C* is the concentration of solute Mn atom in Al matrix, and can be estimated using *EC* by neglecting the contribution of Fe and Si on *EC* due to their low solubility with the following equation [27]:

(4) $$C=(\frac{1.74}{EC}-0.0317)/0.033$$

**Figure 6:**
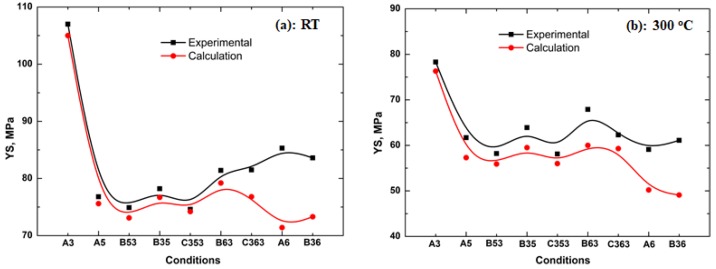
Experimental and calculated YS at RT (**a**) and 300°C (**b**) after various heat treatments.

With the above equations and characters of dispersoids in Table 2, the contribution of solid-solution atoms and dispersoids on YS can be estimated. The differences in YS (ΔYS) between A3 and other heat treatments were calculated in present work, and the comparison between calculated data and the experimental data is shown in Figure 6. It can be seen that the tendency of evolution of YS at both RT and 300 °C is the same between experimental and calculation data. However, the match between experiment and calculation is closer at RT (Figure 6a) than at 300 °C (Figure 6b), which can be attributed to the more active planes and free paths for the movement of dislocation at 300 °C. Also, a bigger difference between calculated and experimental data is observed in the conditions of A6 and B36, in which the Mn is fully solute in the matrix at higher temperature (625 °C) at both RT and 300 °C, which are the last two points in Figure 6. The reason for this is unclear, but it is likely due to the formation of “solute drag” from Mn and Mg atoms [39,40,41], which hinders the movement of dislocation and leads to a further increase of strength.

### 3.3. Creep Resistance

**Figure 7:**
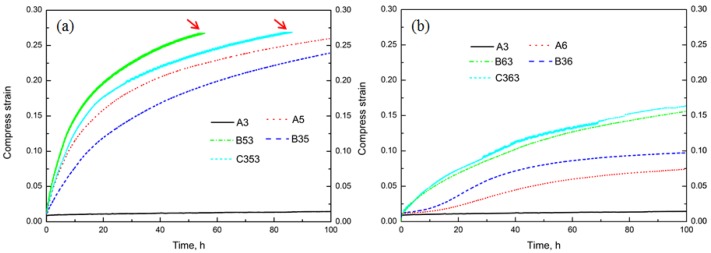
Creep curves after various heat treatments involved at 500 °C (**a**) and 625 °C (**b**).

In our previous study [9], it was found that dispersoids have great effect on creep resistance. The creep tests at 300 °C after heat treatments were also performed in the present work; results are shown in Figure 7. This shows that the total creep strain is greatly related to the heat treatment. Generally, the total strain treated at 625 °C is much less than 500 °C, such as the B53 in Figure 7a and B63 in Figure 7b. It can be observed that the creep strain has reached the limitation of the creep machine (indicted by the red arrow in Figure 7a, also in C353) after 55 hours in B53, but is only 0.152 in B63 after 100 h, showing the different role of solute atoms and dispersoids on the creep property. The creep strain seems lower after heat treatment with lower temperature (375 °C) in the first step, such as B35 and B53 in Figure 7a, B36 and B63 in Figure 7b, which is similar to the evolution of YS shown in Table 3 and Figure 6. Furthermore, though the highest YS at 300 °C is obtained in A6 (Table 3), the minimum creep strain is obtained in A3, which has the finest but the highest volume fraction of dispersoids, further confirming the significant positive influence of dispersoids on creep resistance.

**Figure 8:**
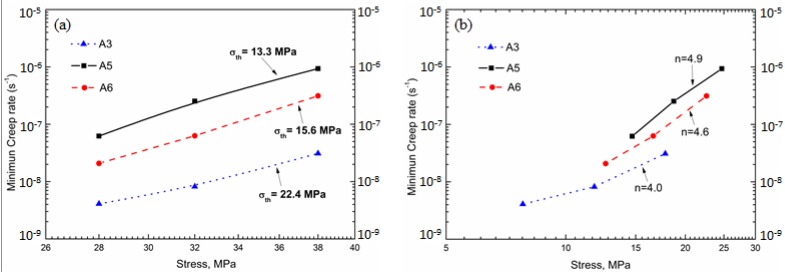
Threshold stress *σ*_th_ (**a**) and stress exponent n (**b**) in A3, A5 and A6.

According to the compression creep condition applied in the present work, the creep mechanism is primarily controlled by the glide and climb of dislocations during creep [42]. However, the difference between various heat treatments is hard to distinguish. The threshold stress *σ*_th_ and true stress exponent n are reported to be two significant parameters to characterize creep resistance. Therefore, A3, A5, and A6 were performed for the step-load creep test. The threshold stress *σ*_th_ is calculated as the stress level when the minimum strain rate (ε) is extrapolated to 10^−10^ s^−1^ and true stress exponent is the slope of lnε − ln(*σ* − *σ*_th_) curve [43]; results are shown in Figure 8.

As shown in Figure 8a, *σ*_th_ increases in the order of A5 (13.3 MPa) to A6 (15.6 MPa), and further to A3 (22.4 MPa), indicating the important role of dispersoids (A3) and solute atoms (A6) in creep resistance. Though the difference between threshold stress after various heat treatment is not so big, an increase of ~3MPa in threshold stress can be translated into an order of magnitude decrease in minimum creep according to the literature [44]. In the present work, the difference of threshold stress between A6 (15.6 MPa) and A5 (13.3 MPa) is 2.3 MPa, but a minimum creep rate 7 times lower in A6 than A5 was observed in Figure 8a. The true stress exponent n is calculated to be in the range of 4–5 (Figure 8b), which indicates that creep is controlled by the dislocation glide or/and climb at elevated temperature [45,46], confirming the dislocation-control creep mechanism in the present work.

Since creep is confirmed to be controlled by the dislocation glide and/or climb, the factors which can affect the movement of dislocation will influence creep behavior, such as the grain boundary, intermetallics, dispersoids, and solute atoms in the present work. From the EBSD measurements, the grain size is found to be similar after all heat treatments, which is in the range of 340–360 μm; the intermetallics also show a similar volume fraction (3.8–4.2 vol. % measured with image analysis). Therefore, the difference in creep resistance, such as *σ*_th_, is mainly controlled by the character of dispersoids and the solute level of atoms.

According to the literature, the contribution of dispersoids to *σ*_th_ can be estimated by the Orowan stress model in the following equation [35]:

(5) $$\sigma_{dispersoid}=\frac{Gb}{4r}{\left(\sqrt{\frac{\pi }{4f}}-1\right)}^{-1}$$

where *G* is the temperature-dependent matrix shear modulus (given as *G* = 25.4[1 − 0.5(*T* − 300)/933] [35]), and *γ* and *f* are the radius and volume fraction of dispersoids in Table 2, respectively. 

On the other hand, the influence of solute atoms *σ*_th_ can be expressed in the equation [47]:

(6) $$\sigma_{solute\;atom}=KGC\exp(\frac{Q}{RT})$$

where *K* is a constant, *G* is the temperature-dependent matrix shear modulus (given as *G* = 25.4[1 − 0.5(*T* − 300)/933] [35]), *C* is the concentration of solute atom in Al matrix, R is the gas content (8.314 JK ^−1^ mol ^−1^), and *T* is the absolute temperature (K). *Q* is the interaction energy between solute atom and dislocation, which can be calculated as follows [48]:

(7) $$Q=2G\frac{1+v}{1-v}(\gamma_{1}-\gamma_{0})\gamma_{0}^{2}$$

where *ν* is the poison ratio (0.34) and *γ*_0_ and *γ*_1_ are the atomic radius of matrix and solute. 

Similar to the evolution of YS, the difference of *σ*_th_ between A3, A5, and A6 was calculated: 6.6 MPa between A3 and A6, and 2.3 MPa between A3 and A5, which is close to the experimental data (6.8 MPa between A3 and A6 and 2.3 MPa between A3 and A5), indicating the applicability of the model to predict creep behavior of 3004 alloy in the present work. Therefore, the creep threshold stress *σ*_th_ in other heat treatments was also calculated using this model; results are shown in Table 4. 

It can be seen in Table 4 that *σ*_th_ varies with heat treatments though with minor differences, and the tendency of evolution is similar to the creep curves shown in Figure 7. For instance, the total creep strain increases from B35 to C353 and further to B53 (Figure 7a), while the creep threshold stress also decreases in the same order: B35 (13.4 MPa) to C535 (13.1 MPa) and then to B53 (12.8 MPa). In addition, it can be observed that the temperature of first-step treatment plays significant role in the evolution of threshold stress. Similar to the evolution of YS, the creep threshold stress with lower temperature in first-step heat treatments is higher than with higher temperature, which is indicated by the higher creep threshold stress in B35 and B36 than in B53 and B63, respectively. 

## 4. Conclusions

(1) Coarsening of dispersoids takes place with increasing temperature from 375 °C to 500 °C, while the dissolution of dispersoids happens by further increasing the temperature to 625 °C. The behavior including size and distribution of dispersoids, as well as the PFZ during various heat treatments, have been fully characterized. 

(2) The evolution of elevated-temperature strength and creep resistance is greatly related to the temperature of each step during the multi-step heat treatments. The temperature of the first-step heat treatment plays a significant role, i.e., higher alloy properties can be obtained with lower-temperature heat treatment as the first step, while they decrease with increasing temperature in the last-step. 

(3) The creep behavior under all the studied heat treatments is defined as dislocation-controlled mechanism with the stress exponent between 4 and 5, but the creep threshold stress is varying with the heat treatment, which is calculated to be 13–23 MPa. 

(4) Suitable models have been preliminarily established to relate the evolution of strength and creep resistance to the behavior of dispersoids and solid-solution levels of elements during the studied multi-step heat treatments.

## Figures and Tables

**Table 1:** Parameters of heat treatments used in this study.

Code	Parameter	Code	Parameter
A3	375 °C/48 h	-	-
A5	500 °C/10 h	A6	625 °C/4 h
B53	500 °C/10 h + 375 °C/48 h	B63	625 °C/4 h + 375 °C/48 h
B35	375 °C/48 h + 500 °C/10 h	B36	375 °C/48 h + 625 °C/4 h
C353	375 °C/48 h + 500 °C/10 h + 375 °C/48 h	C363	375 °C/48 h + 625 °C/4 h + 375 °C/48 h

Note: “A” means single-step heat treatment, while “B” and “C” stands for two-step and three-step heat treatment. “3”, “5”and “6” represent the heat treatment of “375 °C/48 h”, “500 °C/10 h”, and “625 °C/4 h”.

**Table 2:** Characteristics of dispersoids and PFZ during heat treatments.

Code	Dispersoids		PFZ	Code	Dispersoids		PFZ
	Equivalent diameter nm	Volume fraction vol. %	Volume fraction vol. %		Equivalent diameter n	Volume fraction vol. %	Volume fraction vol. %
A3	67(11) *	2.95(0.68)	28(3)				
A5	107(14)	1.58(0.52)	32(4)	A6	102(12)	0.27(0.13)	–
B53	112(15)	1.36(0.62)	55(8)	B63	82(15)	0.87(0.15)	10(5)
B35	105(12)	1.67(0.54)	37(6)	B36	106(14)	0.51(0.21)	–
C353	111(21)	1.61(0.48)	45(7)	C363	83(12)	1.21(0.15)	8(6)

* Note: standard deviation is shown in bracket.

**Table 3:** YS and EC after various heat treatments.

Code	EC (%IACS)	YS (RT) (MPa)	YS (300 °C) (MPa)	Code	EC (%IACS)	YS (RT) (MPa)	YS (300 °C) (MPa)
A3	37.8 (0.3) *	107.9(3.5)	78.3(0.6)				
A5	39.2(0.5)	77.7(2.3)	61.7(0.9)	A6	26.9 (0.8)	86.2(1.8)	59.3(1.6)
B53	38.1(0.6)	75.8(1.8)	58.2(0.5)	B63	38.3(0.5)	82.3(2.5)	60.9(1.4)
B35	38.9(0.5)	79.1(2.1)	63.9(1.1)	B36	27.9(0.5)	84.5(2.1)	61.9(1.9)
C353	37.9(0.2)	76.4(2.6)	58.6(0.5)	C363	38.1(0.6)	82.4(1.9)	62.3(1.5)

* Note: standard deviation is shown in bracket.

**Table 4:** Calculated creep threshold stress (*σ*_th_) during heat treatments.

Code	B35	B53	C353	B36	B63	C363
*σ*_th_ (MPa)	13.4	12.8	13.1	15.3	14.2	14.5
